# Current News

**Published:** 1891-01

**Authors:** 


### Current News.

### TO THE PRACTITIONERS OF DENTISTRY THROUGHOUT THE WORLD.

The Southern and the American Dental Associations, at their respective annual meetings held during the past summer, by *unanimous* vote of each body declared in favor of utilizing the great occasion of the World's Columbian Exposition, to be held in Chicago in 1893, by organizing a world's meeting of the practitioners of dentistry, to be held at the same time and place, August 31, to September 10, 1893.

In pursuance of this resolution a committee of five from each body was appointed, and the joint committee so created was authorized to add five more to its membership, thus constituting an Executive Committee, clothed with full power to take such action as in its judgment it should deem best to carry out the objects of its organization, such action to be final and binding.

This Executive Committee is composed as follows:

APPOINTED BY THE SOUTHERN DENTAL ASSOCIATION.

L. D. Carpenter, Atlanta, Ga.; J. Y. Crawford, Nashville, Tenn.; W. J. Barton, Paris, Tex.; J. Taft, Cincinnati, Ohio; C. S. Stockton, Newark, N. J.

APPOINTED BY THE AMERICAN DENTAL ASSOCIATION.

L. D. Shepard, Boston, Mass.; W. W. Walker, New York City, N. Y.; A. O. Hunt, Iowa City, Iowa; H. B. Noble, Washington, D. C.; Geo. W. McElhaney, Columbus, Ga.

ELECTED BY JOINT COMMITTEE.

J. C. Storey, Dallas, Tex.; M. W. Foster, Baltimore, Md.; A. W. Harlan, Chicago, Ill.; C. S. Marshall, Chicago, Ill.; J. H. McKellops, St. Louis, Mo.

The Executive Committee have elected the following as its officers:

President, W. W. Walker; Secretary, A. O. Hunt; Treasurer, C. S. Marshall.

It has also adopted a constitution for its government, and decided upon the future organization of the following committees:

1. A Finance Committee.
2. A Programme Committee.
3. A Committee on Exhibits.
4. A Committee on Transportation.
5. A Committee on Reception.
6. A Committee on Registration.
7. A Committee on Printing.
8. A Committee of Conference with State and Local Societies.
9. A Committee on Dental Legislation in this and other countries.
10. An Auditing Committee.
11. A Committee on Invitation.
12. A Committee on Membership.
13. A Committee on Educational and Literary Exhibit.
14. A Committee on Clinics in Operative Dentistry and Oral Surgery.
15. A Committee on Prosthetic Dentistry.

The objects had in view by the two great representative bodies authorizing and inaugurating the movement for the holding of

THE WORLD'S COLUMBIAN DENTAL MEETING

are thus broadly and tersely set forth by the Executive Committee:

"The bringing together for professional, scientific, and social purposes the dentists of the United States and of all other countries."

It is the desire, as it will be the effort, of the Executive Committee to so justify the trust reposed in it that the contemplated meeting shall prove a brilliant and long-to-be-remembered success. It stands pledged to do whatever may be in its power to make the occasion contribute notably to the elevation of the profession, to stimulate the spirit of research, to strengthen fraternal courtesy, and to promote cordial co-operation among all who desire the advancement of dental science and art. The aim would be too limited, if it included less than this, and the enterprise will be a failure if it fall short of a realization of this ambition.

For the accomplishment of these objects the hearty assistance of every college Faculty, of every State and local society, and of every reputable dental practitioner is earnestly solicited.

All practising dentists outside of the United States are cordially invited to participate in the meeting, without contributory cost; and there is reason to believe that the invitation will be accepted by large numbers of foreign dentists.

We desire and expect the presence of distinguished representative dentists from almost every civilized country, and nearly all of note in our own country, resulting in the largest gathering of dental practitioners in the history of the world.

Information as to the progress of the work will be communicated through the journals from time to time, and, meanwhile, every dentist is urged to do all in his power to forward the objects sought.

In behalf of the Executive Committee,

W. W. WALKER,
*President.*

67 WEST NINTH STREET, NEW YORK CITY.

---

NEW JERSEY EXAMINATIONS.—The New Jersey Dental Commission will hold its next meeting for examinations in Orange, N. J., on Tuesday, January 20, 1891.

Persons intending to begin the practice of dentistry in New Jersey must make application to the secretary of the Board prior to January 6, 1891.

G. CARLETON BROWN,
*Secretary.*

ELIZABETH, N. J., December 12, 1890.

